# Supplementary material for: Neuromedin U signaling regulates retrieval of learned salt avoidance in a C. elegans gustatory circuit
Source: Nat Commun. 2020 Apr 29;11:2076. doi: 10.1038/s41467-020-15964-9 (PMC7190830; doi:10.1038/s41467-020-15964-9)
Supplement: Supplementary file 1 — Supplementary Information [file 41467_2020_15964_MOESM1_ESM.pdf]

Supplementary Information for:

Neuromedin U signaling regulates retrieval of learned salt avoidance in a *C. elegans* gustatory circuit

Watteyne et al.

Supplementary Figure 1

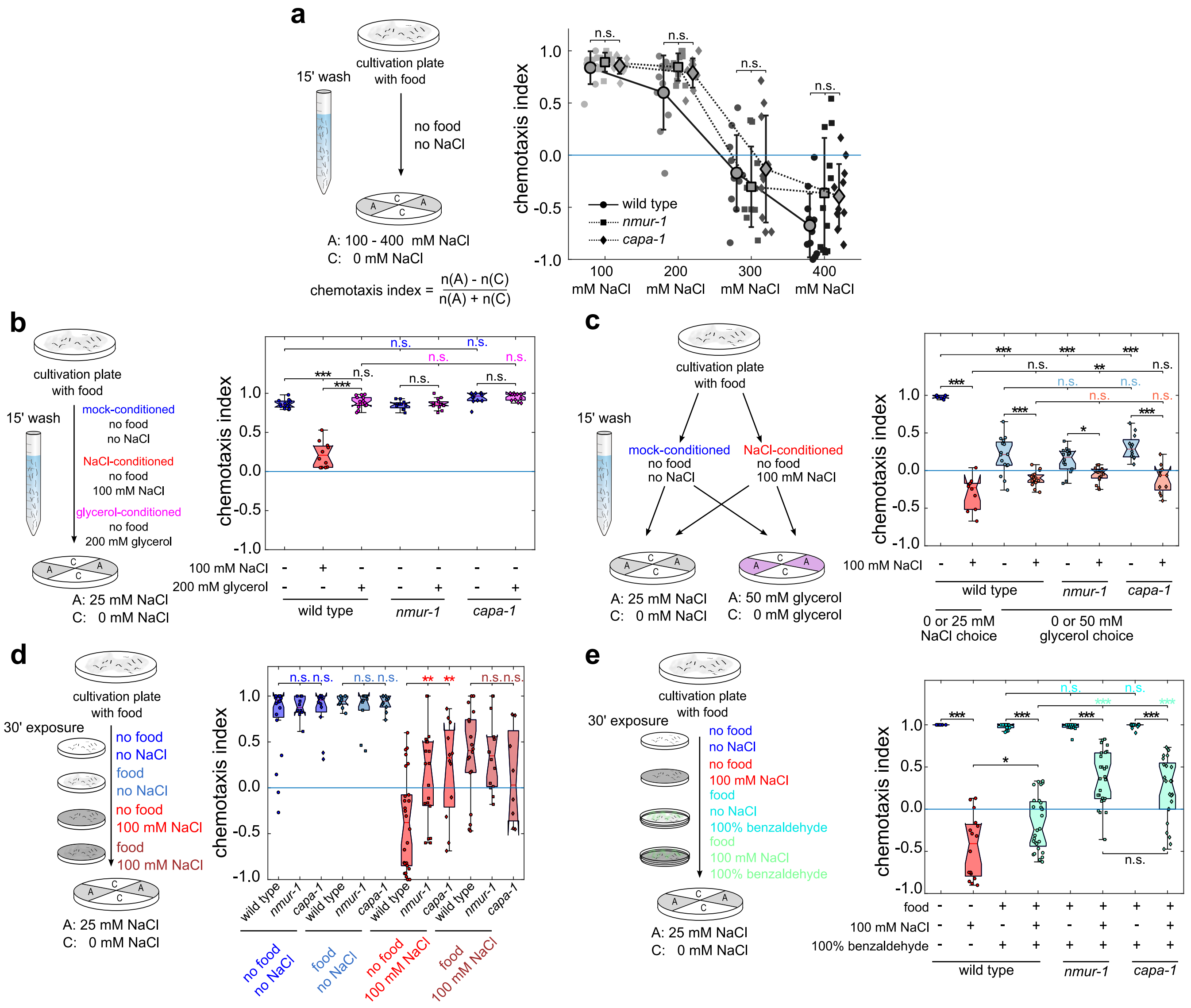

Supplementary Fig. 1. Mutants of *nmur-1* and *capa-1* are defective in associating salt with aversive experience (related to Fig. 1 and Fig. 5)

(a) Chemotaxis behavior of wild type, *nmur-1 (ok1387)* and *capa-1 (ok3065)* mutants to increasing NaCl concentrations. Synchronized adult *C. elegans* are washed for 15 min in NaCl-free buffer. Salt chemotaxis behavior is then assayed on quadrant plates of which two opposing quadrants are supplemented with 100, 200, 300 or 400 mM NaCl. The distribution of animals on the quadrants is determined at 10 min and the chemotaxis index calculated. Wild-type, *nmur-1* and *capa-1* animals are attracted to low concentrations of salt (100 and 200 mM NaCl) but avoid higher concentrations (300 and 400 mM NaCl). From left to right n = 9, 9, 9, 10, 10, 10, 11, 10, 10, 11, 12, 11assays per condition. Error bars represent S.D. **(b)** NaCl chemotaxis behavior of wild-type, *nmur-1 (ok1387)* and *capa-1 (ok3065)* animals after 15 min pre-exposure to 200 mM glycerol in the absence of food. Fed synchronized adult *C. elegans* are washed for 15 min in buffer with (glycerol-conditioned) or without glycerol (mock-conditioned). Wild-type worms washed with 100 mM NaCl buffer (NaCl-conditioned) are used as a control. After washing, NaCl chemotaxis is assayed on quadrant plates of which two opposing quadrants are supplemented with 25 mM NaCl. Glycerol-conditioned wild-type, *nmur-1* and *capa-1* animals retain NaCl attraction. From left to right n = 14, 12, 16, 12, 14, 12, 12 assays per condition. **(c)** Chemotaxis to 50 mM glycerol of wild-type, *nmur-1 (ok1387)* and *capa-1 (ok3065)* animals after 15 min pre-exposure to 100 mM NaCl in the absence of food. Synchronized adult *C. elegans* are washed for 15 min in NaCl-free buffer (mock-conditioned) or in 100 mM NaCl buffer (NaCl-conditioned). Chemotaxis behavior to glycerol is then tested on quadrant plates in which 50 mM glycerol is added to two opposing quadrants. Quadrant plates containing 25 mM NaCl are used as control. Mock-conditioned wild-type, *nmur-1* and *capa-1* animals show weak attraction to 50 mM glycerol, which is slightly reduced after NaCl conditioning for all genotypes. From left to right n = 8, 10, 16, 17, 18, 18, 12, 12 assays per condition. **(d)** Salt chemotaxis behavior of wild-type, *nmur-1 (ok1387)* and *capa-1 (ok3065)* animals after conditioning with NaCl in the presence or absence of bacteria (food). Synchronized adult *C. elegans* are conditioned for 30 min on agar plates supplemented with 100 mM NaCl. Conditioning plates without NaCl and/or seeded with *E. coli* OP50 bacteria are used as a control. After conditioning, NaCl chemotaxis behavior is assayed on quadrant plates in which two opposing quadrants contain 25 mM NaCl. Mock-conditioned animals, which have not been pre-exposed to salt (with or without food) show strong attraction to NaCl. Conditioning with salt in the absence of food switches salt chemotaxis behavior to an avoidance response, but this conditioned response is significantly reduced in *nmur-1* and *capa-1* mutants. *nmur-1* and *capa-1* animals have normal salt chemotaxis behavior when conditioned with salt in the presence of food, which indicates that they are defective in gustatory aversive learning. From left to right n = 20, 10, 10, 10, 10, 10, 26, 19, 12, 18, 14, 8 assays per condition. **(e)** Salt chemotaxis behavior of wild-type, *nmur-1 (ok1387)* and *capa-1 (ok3065)* animals after conditioning with NaCl and aversive concentrations of benzaldehyde. Synchronized adult *C. elegans* are pre-exposed to 100% benzaldehyde for 30 min on seeded agar plates with or without 100 mM NaCl. Unseeded conditioning plates with or without NaCl are used as a control. After conditioning, salt chemotaxis behavior is assayed on quadrant plates in which two opposing quadrants contain 25 mM NaCl. Wild-type animals avoid NaCl when pre-exposed to it in the presence of benzaldehyde. This conditioned response is impaired in mutants of *nmur-1* and *capa-1*. From left to right n = 8, 16, 18, 18, 17, 28, 26, 25 assays per condition. Two-way ANOVA followed by Tukey's multiple comparison test used to determine statistical significances in (a-e). n.s. not significant; *$p \leq 0.05$; **$p \leq 0.01$; ***$p \leq 0.001$.

Boxplots show medians, 25th and 75th percentiles as box limits. The whiskers extend to the most extreme data points not considered as outlier.

Supplementary Figure 2

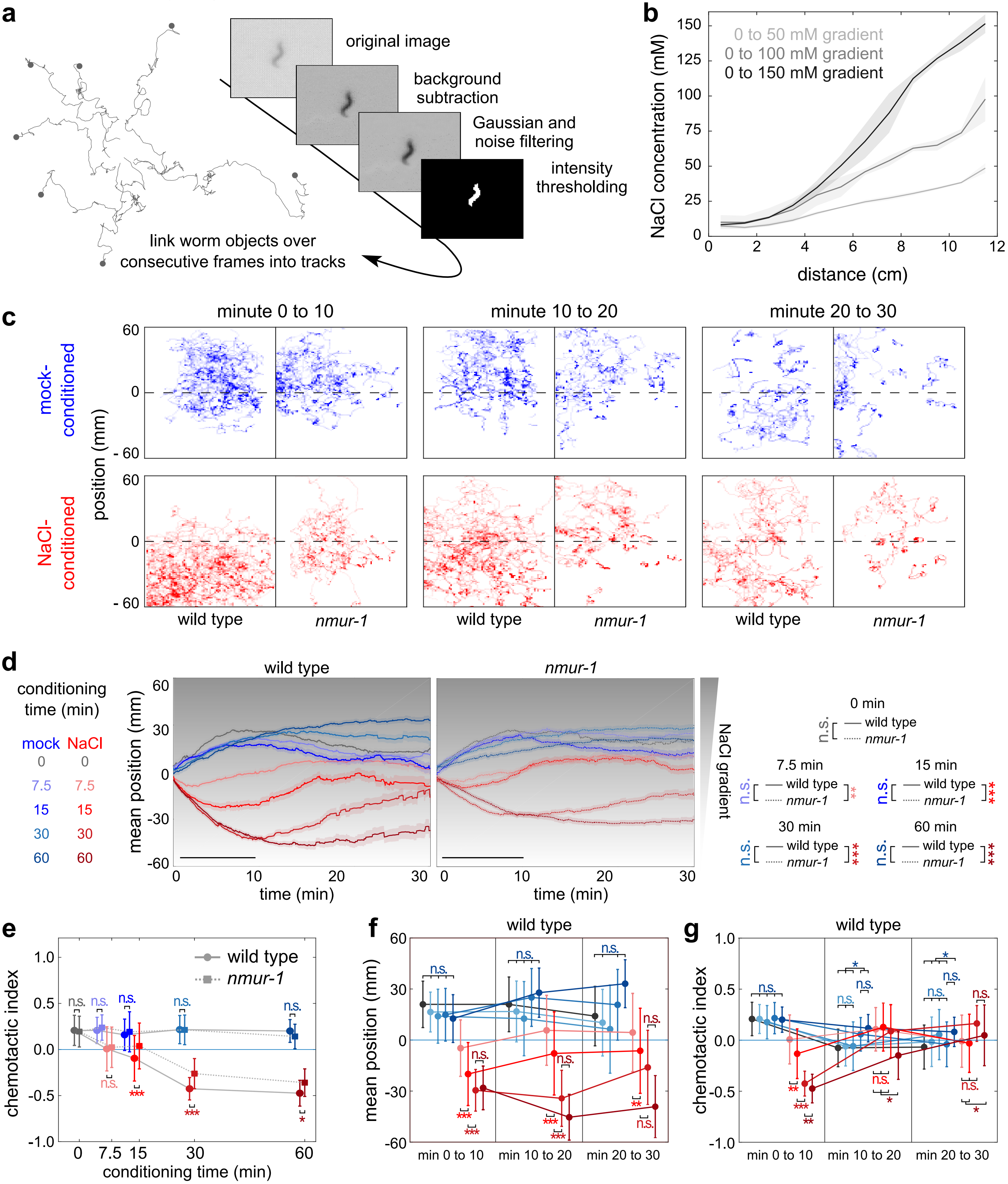

Supplementary Fig. 2. Mutants of *nmur-1* are impaired in gustatory aversive learning with increased conditioning times (related to Fig. 3)

(a) Tracking of salt chemotaxis behavior on linear NaCl gradients. Worm populations are imaged at 2 frames per second while navigating 12 x 12 cm square agar Petri plates. On each frame, individual worms are identified by background subtraction (background computed as average of 50 frames evenly spaced throughout the image sequence), followed by Gaussian and noise filtering of the foreground image. The frame is then binarized into worm objects or background by intensity thresholding. Finally, individual worms are linked into tracks over consecutive frames. **(b)** Linear salt gradients are generated in 12 x 12 cm square agar Petri plates. After casting the plates and allowing NaCl to diffuse for 24 hours, the linear relationship of NaCl concentrations along the y-axis is measured using a chloride electrode. 1 cm wide samples along the y-axis, n = 3 independent preparations. **(c)** Navigation trajectories of 25 – 50 *C. elegans* wild-type and *nmur-1 (ok1387)* animals after mock- and NaCl-conditioning. Panels display the relative x-y residence on 0 to 100 mM linear NaCl gradients in 10-min intervals. **(d - e)** NaCl chemotaxis behavior of wild-type and *nmur-1 (ok1387)* animals on a 0 – 100 mM NaCl gradient after mock- or NaCl-conditioning for various durations (0, 7.5, 15, 30 or 60 min). (d) Mean position on the NaCl gradient through time, delineated per genotype. Blue and red shading darkens with increased conditioning time. Black horizontal bars indicate the time interval used for statistical comparison of the mean position on the gradient and calculating the chemotactic index. (e) Average chemotactic index with increasing mock- and NaCl-conditioning time. NaCl attraction is retained with increased mock-conditioning time in both wild-type and *nmur-1* animals. Wild-type worms show progressive changes in learned salt aversion with increased NaCl-conditioning time whereas *nmur-1* worms show a learning defect as compared to wild type from 15 min NaCl-conditioning onwards. Source data provided as a source data file. From left to right n = 51, 73, 73, 65, 90, 74, 65, 89, 50, 64, 91, 90, 61, 72, 73, 50, 82, 78 animals per condition. (**f - g**) NaCl chemotaxis behavior quantified in three sequential 10-min time intervals. Data from wild type in panel d. In each time interval, (f) the mean position and (g) chemotactic index is calculated for individual animals after 0, 7.5, 15, 30 or 60 min of conditioning. From left to right n = 51, 73, 71, 91, 73, 90, 57, 61, 82, 117, 81, 87, 86, 85, 82, 67, 45, 30, 56, 56, 79, 57, 52, 55, 34, 25, 20 animals per condition. (f) Mock-conditioned animals show mean positions up the gradient independent of the duration of mock-conditioning, after which they maintain this position over subsequent time windows. Similarly, NaCl-conditioned animals migrate to lower mean positions on the NaCl gradient in the first 10-min time interval, and display gradual displacement back to higher salt concentrations over time. The magnitude of salt avoidance depends on the conditioning time. (g) Chemotactic indices for NaCl-conditioned animals in the first 10-min time interval depend on the duration of conditioning. Indices for both mock- and NaCl-conditioned worms average out to zero in subsequent time windows as worms show less displacement up or down the gradient compared to the first 10-min interval.

Error bars in (e-g) represent S.D. Significances in (d-g) determined by three-way ANOVA followed by Tukey's HSD criterion. *$p \leq 0.05$; **$p \leq 0.01$; ***$p \leq 0.001$. Boxplots show medians, 25th and 75th percentiles as box limits. The whiskers extend to the most extreme data points not considered as outlier.

Supplementary Figure 3

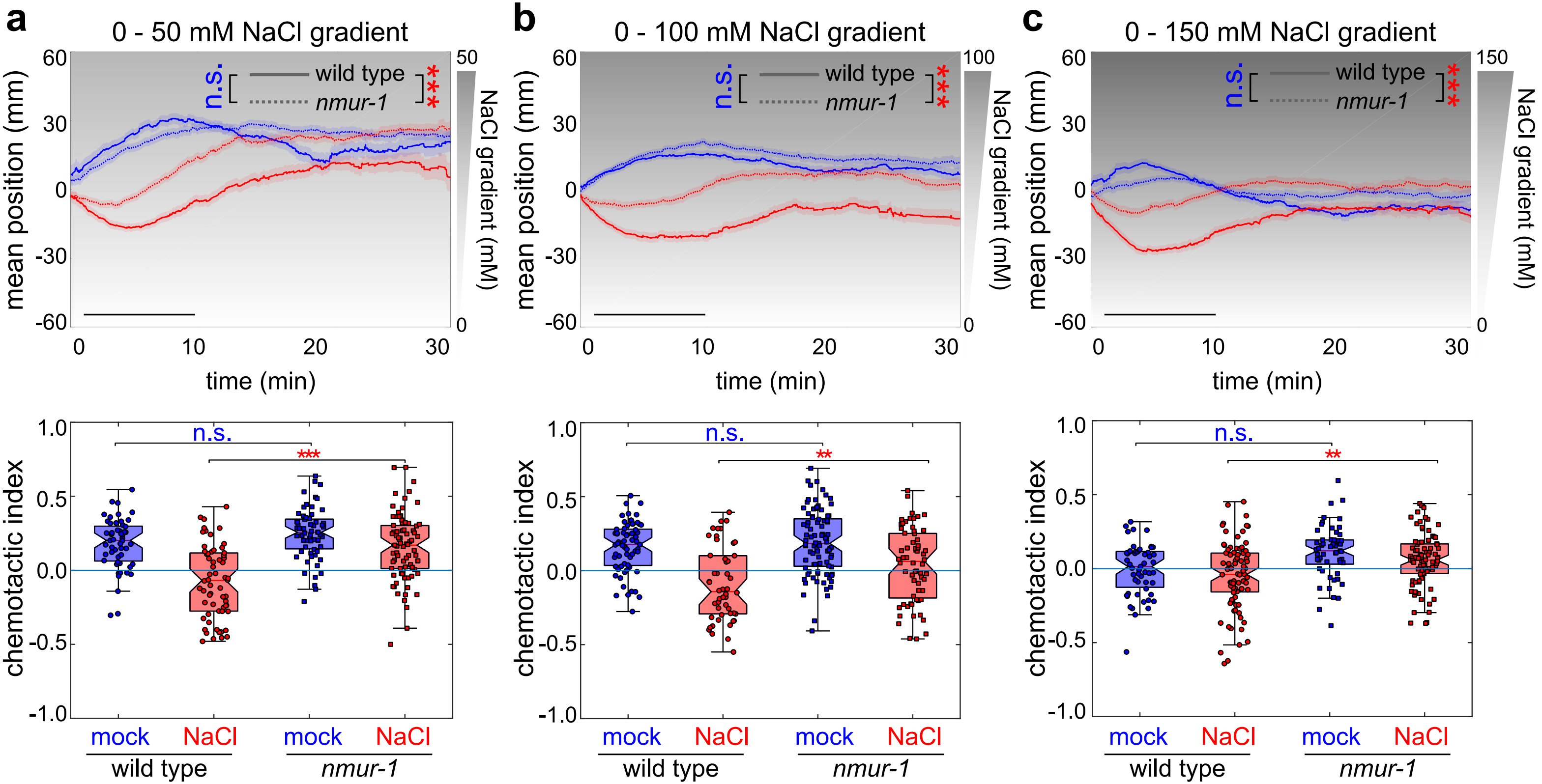

Supplementary Fig. 3. *nmur-1* animals are defective in gustatory aversive learning on different linear NaCl gradients (related to Fig. 3)

(a – c) Average positions and corresponding chemotactic indices of wild-type and *nmur-1 (ok1387)* animals on (a) 0 – 50 mM, (b) 0 – 100 mM and (c) 0 – 150 mM linear NaCl gradients. Black horizontal bars indicate the time interval used for statistical comparison of the mean position on the gradient and for calculating the chemotactic index. Shaded regions represent S.E.M. NaCl-conditioned wild-type worms show learned NaCl aversion while *nmur-1* mutants display a significantly reduced learning ability across different linear NaCl gradients. Significances determined by two-way ANOVA followed by Tukey's HSD criterion. n.s. not significant; $^{**}p \leq 0.01$; $^{***}p \leq 0.001$. Boxplots show medians, 25th and 75th percentiles as box limits. The whiskers extend to the most extreme data points not considered as outlier. (a) From left to right n = 53, 62, 63, 79 animals per condition. (b) From left to right n = 71, 57, 95, 71 animals per condition. (c) From left to right n = 55, 84, 57, 87 animals per condition.

Supplementary Figure 4

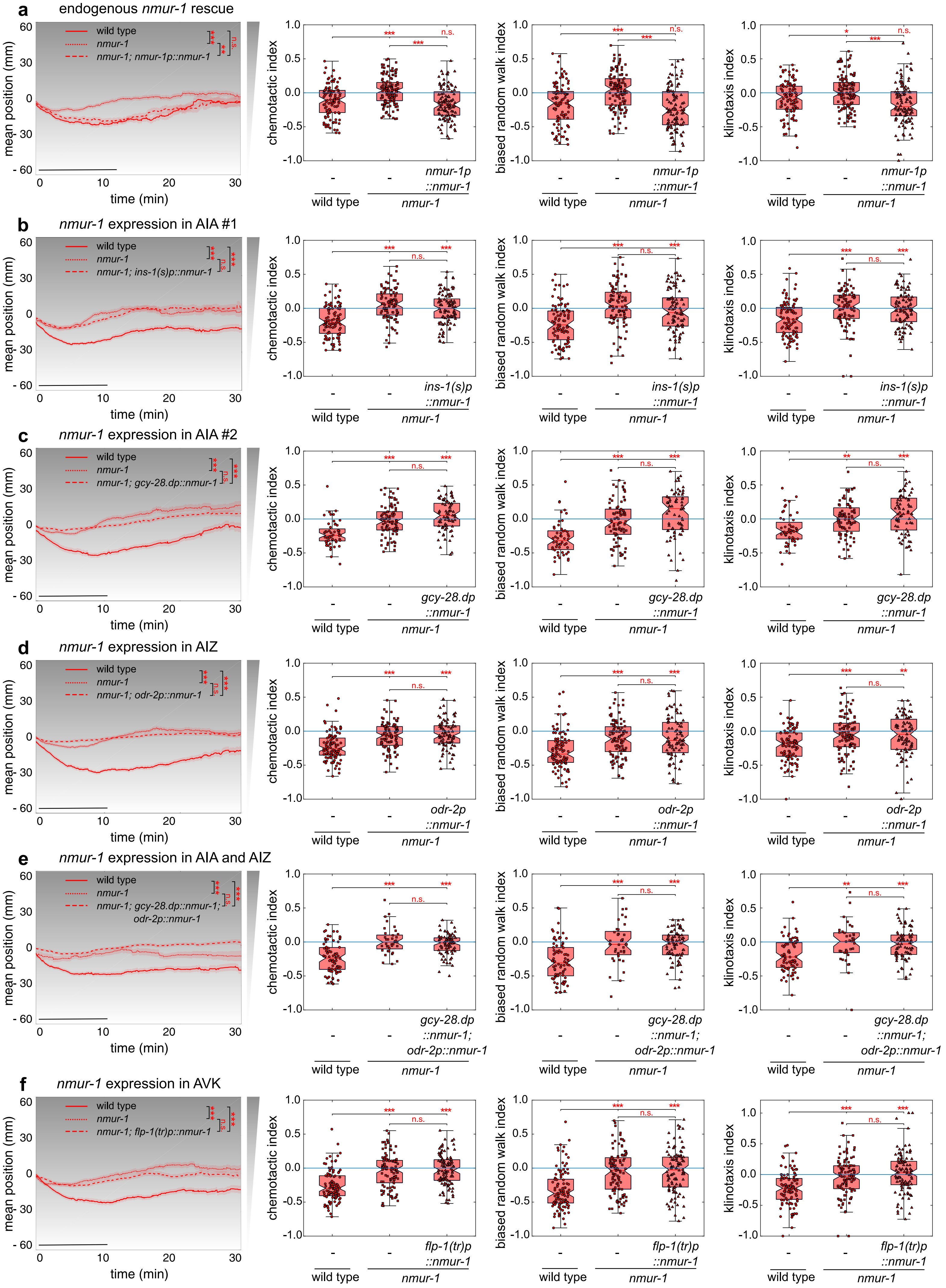

Supplementary Fig. 4. Expression of *nmur-1* in selected interneurons does not rescue learned salt aversion of *nmur-1* animals on linear NaCl gradients (related to Fig. 3f)

Mean position on a 0 – 100 mM NaCl gradient, and chemotactic, biased random walk and klinotaxis indices for NaCl-conditioned wild-type and *nmur-1 (ok1387)* mutants. Learned salt aversion is rescued in *nmur-1* animals expressing wild-type *nmur-1* **(a)** under the control of its endogenous promoter (*nmur-1p::nmur-1*). From left to right n = 103, 127, 110 animals per genotype. Mutant phenotypes are not rescued when expressing *nmur-1* **(b)** in AIA interneurons using the *ins-1(s)* promoter (*ins-1(s)p::nmur-1*[1]), from left to right n = 98, 96, 99 animals per genotype; **(c)** in AIA under control of the *gcy-28d* promoter (*gcy-28dp::nmur-1*[2]), from left to right n = 52, 86, 73 animals per genotype; **(d)** in AIZ interneurons using the *odr-2* promoter (*odr-2p::nmur-1*[3]), from left to right n = 101, 102, 84 animals per genotype; **(e)** in AIA and AIZ interneurons using the *gcy-28d* and *odr-2* promoters (*gcy-28dp::nmur-1*; *odr-2p::nmur-1*), from left to right n = 79, 37, 80 animals per genotype; or **(f)** in AVK interneurons under control of the *flp-1(tr)* promoter (*flp-1(tr)p::nmur-1*[4]), from left to right n = 106, 105, 105 animals per genotype.

Black bar on mean position plot (left panel) indicates the time interval used for statistical comparison of the mean position and for calculating the chemotactic, biased random walk and klinotaxis indices. Shaded area around mean position traces denotes S.E.M. Statistical comparisons by one-way ANOVA and Tukey post-hoc test or two-sided Kruskal-Wallis and Dunn's post-hoc test when not all groups are normally distributed. *$p \leq 0.05$; **$p \leq 0.01$; ***$p \leq 0.001$. Boxplots show medians, 25th and 75th percentiles as box limits. The whiskers extend to the most extreme data points not considered as outlier.

Supplementary Figure 5

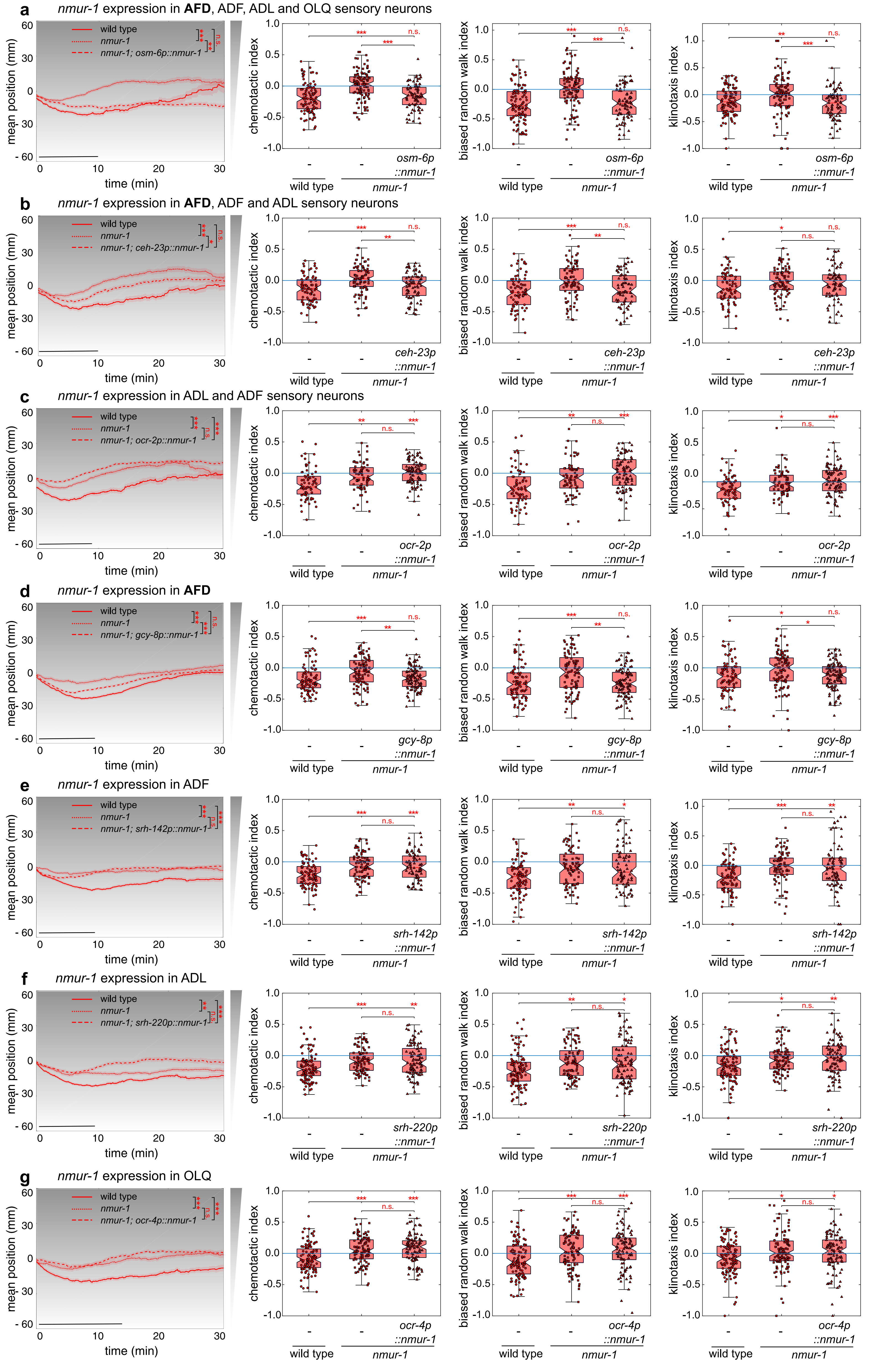

Supplementary Fig. 5. Cell-specific expression of *nmur-1* in AFD, but not in other sensory neurons, rescues *nmur-1* phenotypes on linear NaCl gradients (related to Fig. 3f)

Mean position on a 0 – 100 mM NaCl gradient, and chemotactic, biased random walk and klinotaxis indices for NaCl-conditioned wild type, *nmur-1 (ok1387)* animals, and transgenic mutants in which *nmur-1* expression is specifically restored **(a)** in AFD, ADF, ADL and OLQ sensory neurons under control of the *osm-6* promoter (*osm-6p::nmur-1*[5]), from left to right n = 102, 107, 82 animals per genotype; **(b)** in AFD, ADF and ADL sensory neurons using the *ceh-23* promoter (*ceh-23p::nmur-1*[6]), from left to right n = 81, 88, 75 animals per genotype; **(c)** in ADF and ADL sensory neurons under control of the *ocr-2* promoter (*ocr-2p::nmur-1*[7]), from left to right n = 71, 71, 93 animals per genotype; **(d)** in AFD sensory neurons using the *gcy-8* promoter (*gcy-8p::nmur-1*[8]), from left to right n = 89, 97, 109 animals per genotype; **(e)** in ADF sensory neurons under control of the *srh-142* promoter (*srh-142p::nmur-1*[9]), from left to right n = 90, 87, 80 animals per genotype; **(f)** in ADL sensory neurons using the *srh-220* promoter (*srh-220p::nmur-1*[10]), from left to right n = 112, 94, 99 animals per genotype; and **(g)** in OLQ sensory neurons under control of the *ocr-4* promoter (*ocr-4p::nmur-1*[7]), from left to right n = 112, 109, 101 animals per genotype.

Black bar on mean position plot (left panel) indicates the time interval used for statistical comparison of the mean position on the gradient and for calculating the chemotactic, biased random walk and klinotaxis indices. Shaded area around mean position traces denotes S.E.M. Statistical comparisons by one-way ANOVA and Tukey post-hoc test or two-sided Kruskal-Wallis and Dunn's post-hoc test when not all groups are normally distributed. *$p \leq 0.05$; **$p \leq 0.01$; ***$p \leq 0.001$. Boxplots show medians, 25th and 75th percentiles as box limits. The whiskers extend to the most extreme data points not considered as outlier.

Supplementary Figure 6

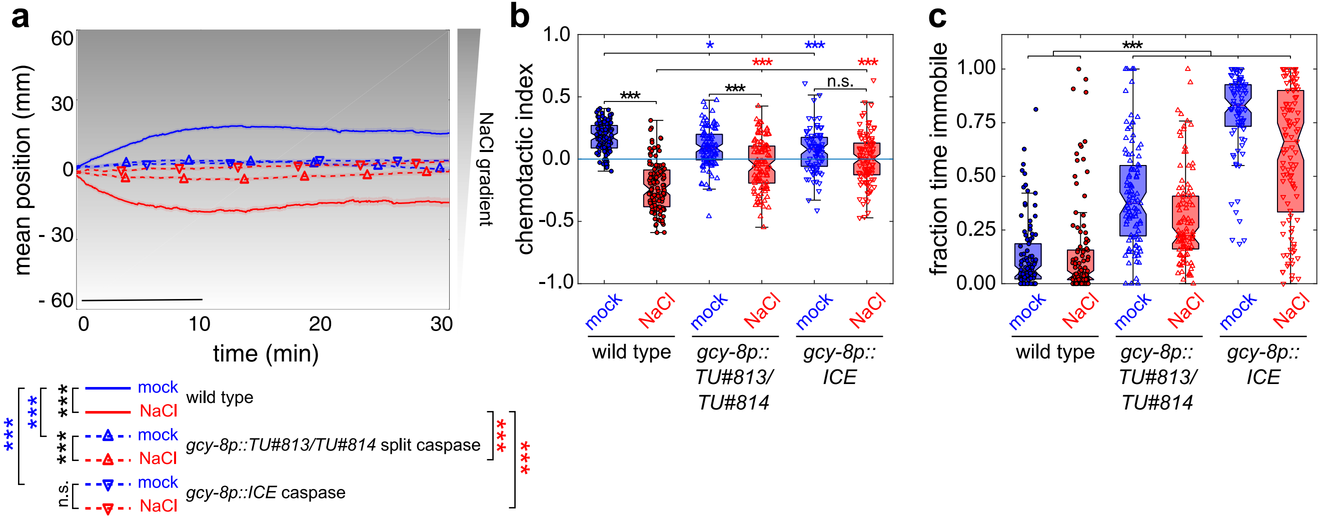

Supplementary Fig. 6. AFD ablation disrupts salt chemotaxis behavior on NaCl gradients

(a-c) Mock- and NaCl-conditioned behavior of two independent transgenic strains in which AFD is genetically ablated (GN112 *gcy-8p::TU#813*; *gcy-8p::TU#814* split caspase expression in AFD[11] and JPS271 *gcy-8p::ICE* human caspase expression in AFD[12]). (a) Mean population position on a 0 – 100 mM NaCl gradient after mock- and NaCl-conditioning. Shaded regions represent S.E.M. Black bar indicates the time interval for statistical comparison of the mean position on the gradient and for calculating (b) the chemotactic index and (c) the average fraction of time spent pausing on the NaCl gradient. From left to right n = 102, 111, 101, 105, 81, 105 animals per condition.

Significances in (a, b) determined by two-way ANOVA with Tukey's HSD post-hoc test. Two-sided kruskal-Wallis with Dunn's multiple comparison test for the fraction of time spent pausing on the NaCl gradient (c). n.s. not significant; $*p \leq 0.05$; $***p \leq 0.001$. Boxplots show medians, 25th and 75th percentiles as box limits. The whiskers extend to the most extreme data points not considered as outlier.

Supplementary Figure 7

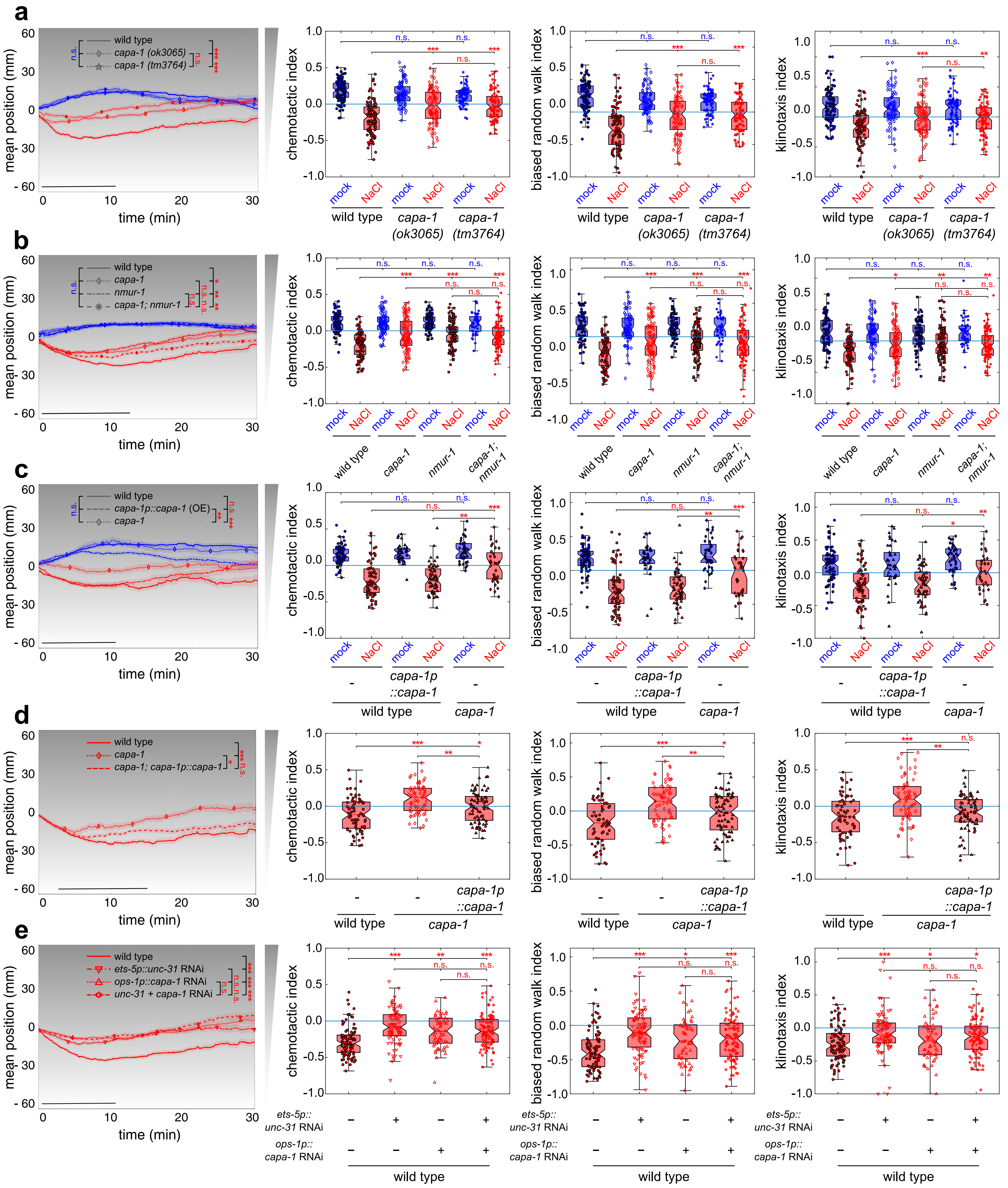

Supplementary Fig. 7. CAPA-1 signaling from ASG neurons regulates the experience-dependent modulation of NaCl chemotaxis behavior (related to Fig. 5)

Mean position on a 0 to 100 mM NaCl-gradient, and chemotactic, biased random walk and klinotaxis indices of mock- and NaCl-conditioned animals for **(a)** two mutant alleles of *capa-1*, *ok3065* and *tm3764*, from left to right n = 105, 102, 87, 93, 86, 97 animals per condition; **(b)** *capa-1 (ok3065)*, *nmur-1 (Ist1672)* and *capa-1 (ok3065); nmur-1 (Ist1672)* double mutants, from left to right n = 77, 86, 81, 95, 83, 100, 48, 101 animals per condition; **(c)** transgenic worms overexpressing *capa-1*, from left to right n = 83, 77, 38, 56, 42, 36 animals per condition; **(d)** NaCl-conditioned *capa-1 (ok3605)* mutant worms in which *capa-1* expression is restored under control of its endogenous promoter (*capa-1p::capa-1*), from left to right n = 94, 83, 88 animals per genotype; **(e)** NaCl-conditioned animals with RNAi knockdown of *unc-31* using the *ets-5* promoter (expressed in ASG and BAG[13]), RNAi knockdown of *capa-1* using the ASG-specific *ops-1* promoter[14], or both, from left to right n = 92, 88, 60, 102 animals per genotype.

Black bar on mean position plot (left panel) indicates the time interval used for statistical comparison of the mean position on the gradient and for calculating the chemotactic, biased random walk and klinotaxis indices. Shaded area around mean position traces denotes S.E.M. Statistical comparisons by one-way ANOVA and Tukey post-hoc test or two-sided Kruskal-Wallis and Dunn's post-hoc test when not all groups are normally distributed in (d-e), or two-way ANOVA with Tukey's HSD post-hoc test in (a-c). n.s. not significant; *$p \leq 0.05$; **$p \leq 0.01$; ***$p \leq 0.001$. Boxplots show medians, 25th and 75th percentiles as box limits. The whiskers extend to the most extreme data points not considered as outlier.

Supplementary Figure 8

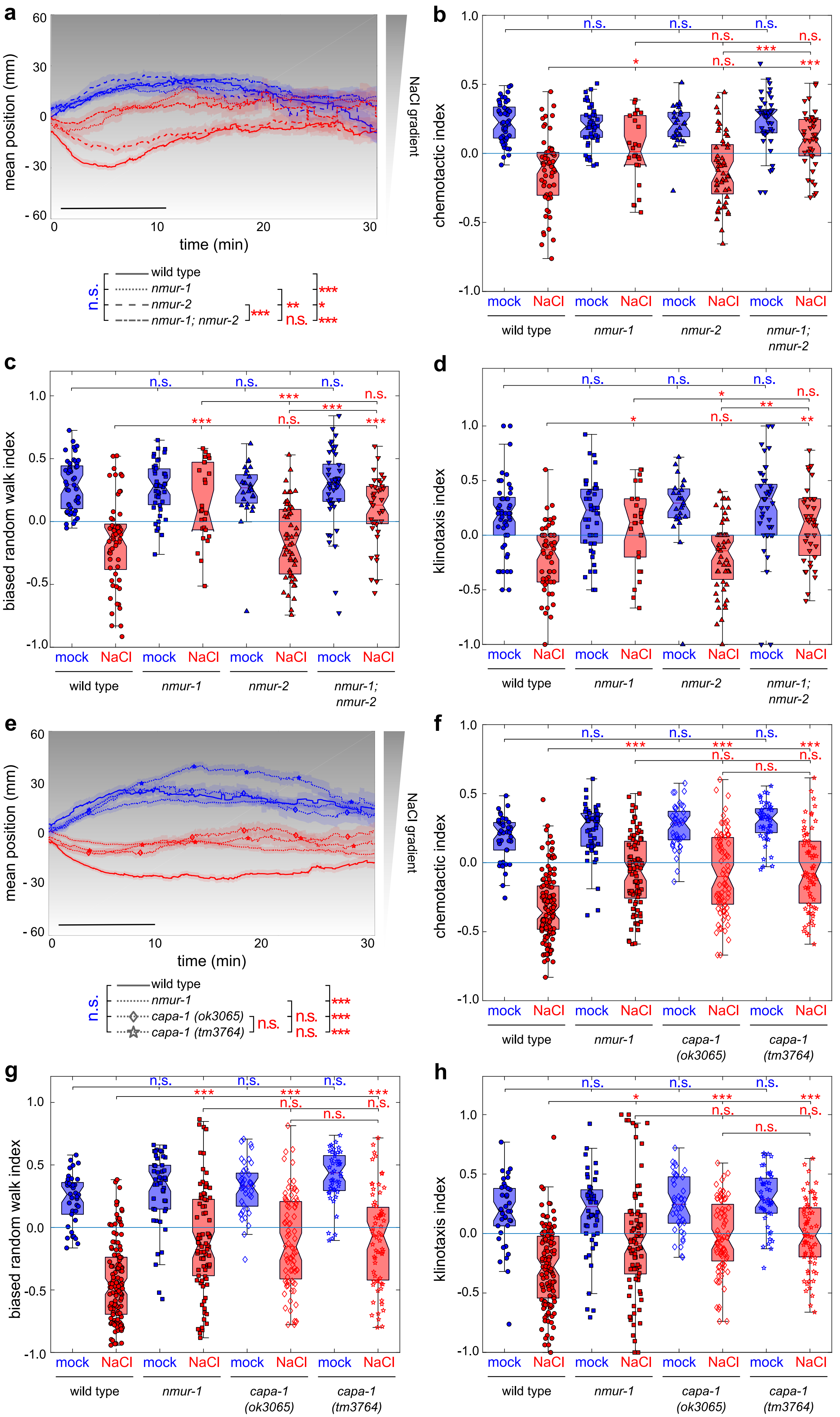

Supplementary Fig. 8. Defects in gustatory aversive learning of CAPA-1 signaling mutants are independent of the bacterial diet and the CAPA-1 receptor NMUR-2

(a) Mean position of *nmur-1 (ok1387)* and *nmur-2 (ok3502)* single and double mutants on a 0 – 100 mM NaCl gradient after mock- and NaCl-conditioning. **(b)** Chemotactic, **(c)** biased random walk and **(d)** klinotaxis indices for wild type, *nmur-1* and *nmur-2* single and double mutants after mock- and NaCl-conditioning. From left to right n = 49, 57, 43, 26, 27, 52, 45, 39 animals per genotype. **(e)** Mean position on a 0 – 100 mM NaCl gradient for mock- and NaCl-conditioned wild type, *nmur-1 (ok1387)*, *capa-1 (ok3605)* and *capa-1 (tm3764)* animals cultivated on *E. coli* HT115 bacteria. **(f)** Chemotactic, **(g)** biased random walk and **(h)** klinotaxis indices for wild-type animals, *capa-1* and *nmur-1* mutants cultivated on *E. coli* HT115. From left to right n = 38, 122, 48, 85, 40, 77, 44, 67 animals per genotype.

Black bar indicates time interval used for statistical comparison of the mean position on the gradient and for calculating the chemotactic, biased random walk and klinotaxis indices. Shaded regions around the mean traces denote S.E.M. in a and e. Statistical comparisons by two-way ANOVA and Tukey's HSD post-hoc. n.s. not significant; *$p \leq 0.05$; **$p \leq 0.01$; ***$p \leq 0.001$. Boxplots show medians, 25th and 75th percentiles as box limits. The whiskers extend to the most extreme data points not considered as outlier.

Supplementary Figure 9

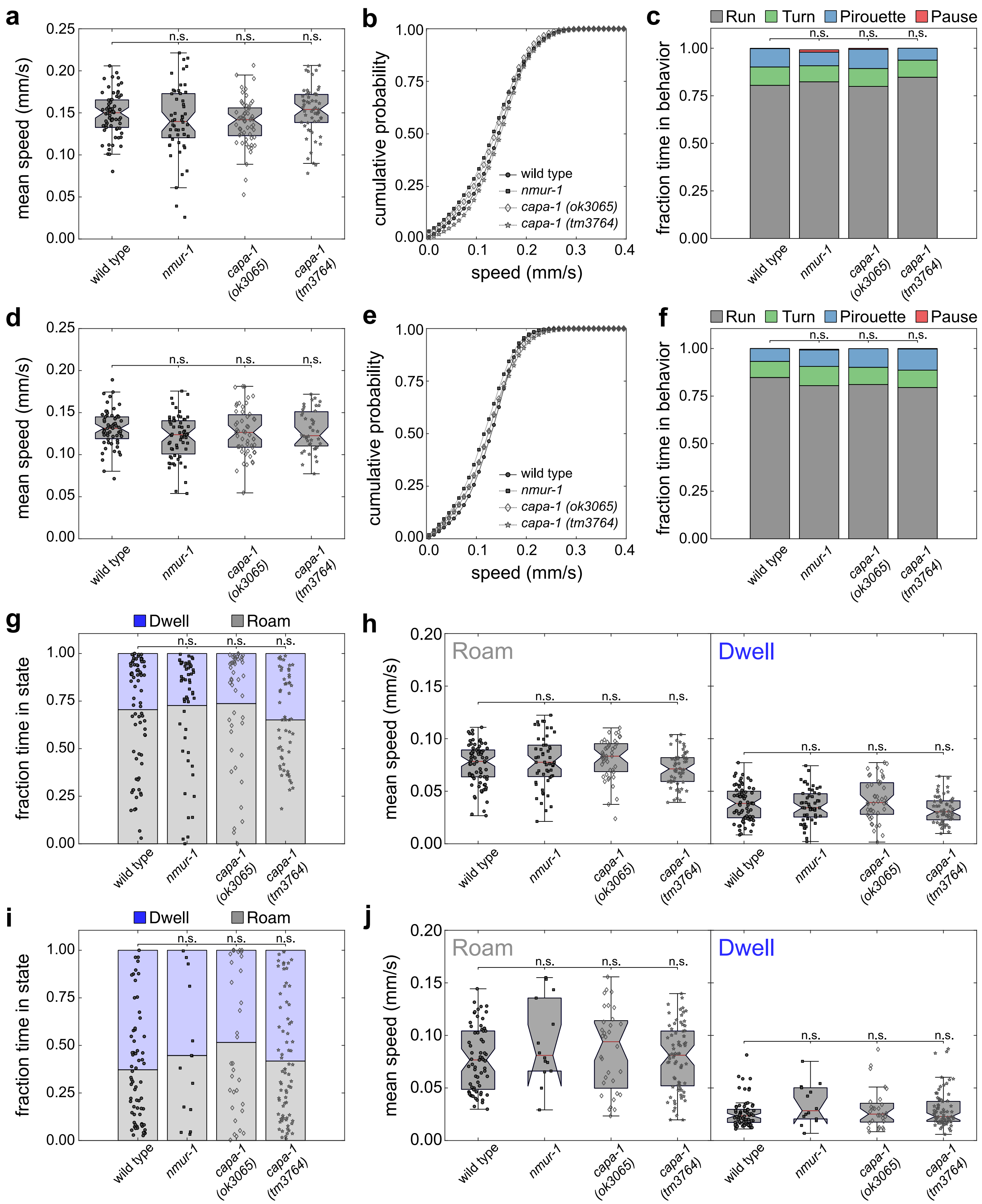

Supplementary Fig. 9. Mutants defective in CAPA-1 signaling show normal food searching behaviors (a - c) Local search behavior of worms grown on *E. coli* OP50. Adult wild-type, *nmur-1 (ok1387)*, *capa-1 (ok3605)* and *capa-1 (tm3764)* animals are removed from OP50 and transferred to an unseeded agar plate for behavioral tracking. (a) The average speed and (b) the cumulative probability distribution of speed for animals after removal from food. (c) Individual worm trajectories are subdivided into discrete stretches of Run (gray) – Turn (green) – Pirouette (blue) or Pause (red) behavior, and average fraction of time in each behavioral state plotted. From left to right n = 66, 52, 57, 56 animals per genotype. **(d - f)** Local search behavior of worms grown on *E. coli* HT115. Adult wild-type, *nmur-1 (ok1387)*, *capa-1 (ok3605)* and *capa-1 (tm3764)* animals are removed from HT115 and transferred to an unseeded agar plate for behavioral tracking. (d) The average speed and (e) the cumulative probability distribution of speed for animals after removal from food. (f) Individual worm trajectories are subdivided into discrete stretches of Run (gray) – Turn (green) – Pirouette (blue) or Pause (red) behavior, and average fraction of time in each behavioral state plotted. From left to right n = 59, 59, 51, 40 animals per genotype. **(g and h)** Quantification of roaming and dwelling behaviors on *E. coli* OP50. While feeding on a bacterial lawn, *C. elegans* alternates between two distinct behavioral states: an active exploratory state referred to as roaming, and more passive behavior termed dwelling. Both states are assigned based on locomotion speed and angular speed (a measure of turning rate). Roaming animals move quickly across the bacterial lawn and turn infrequently to explore the bacterial lawn, while dwelling animals remain in a small area by moving slowly and turning more frequently. (g) The fraction of time spent in the roaming state and (h) average speed during roaming or dwelling for wild type, *nmur-1 (ok1387)*, *capa-1 (ok3605)* and *capa-1 (tm3764)* mutants on OP50. From left to right n = 74, 53, 43, 56 animals per genotype. **(i and j)** Quantification of roaming and dwelling behaviors on *E. coli* HT115. (i) The fraction of time spent in the roaming state and (j) average speed during roaming or dwelling. n = 67, 14, 32, 73 animals per genotype.

Statistical comparisons by one-way ANOVA and Tukey’s post-hoc test or two-sided Kruskal-Wallis with Dunn’s post-hoc test when one of the conditions failed the normality test (a, d, h and j), Chi-square test on binned data (c and f), and two-sided Kruskal-Wallis test followed by Dunn’s post-hoc test (g and i). n.s. not significant. Boxplots show medians, 25th and 75th percentiles as box limits. The whiskers extend to the most extreme data points not considered as outlier.

Supplementary Figure 10

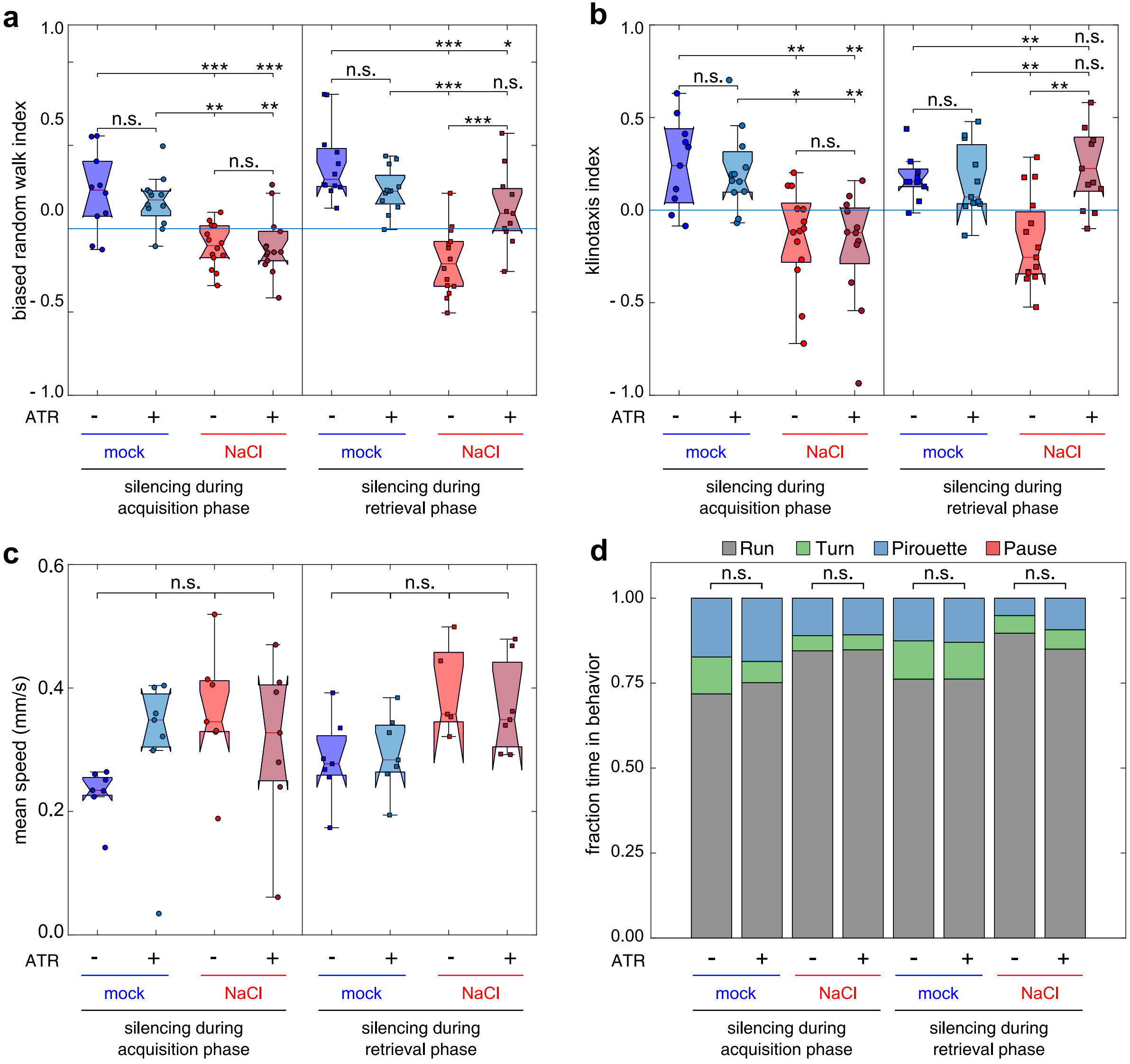

Supplementary Fig. 10. Behavioral effects of silencing CAPA-1 neurons (related to Fig. 7)
The inhibitory opsin Arch is expressed in ASG under control of the *capa-1* promoter. Individual transgenic worms are illuminated with yellow-green light for ASG silencing while being conditioned, or when they are navigating an agar plates with a 0 to 50 mM NaCl linear gradient (a-b) or no gradient (c-d). Arch requires the cofactor *all-trans* retinal (ATR) to be supplemented to the worm culture. Transgenic *C. elegans* not fed ATR-supplemented food serve as a negative control. See Methods for details. **(a)** Biased random walk and **(b)** klinotaxis indices for mock- and NaCl-conditioned animals navigating a 0 to 50 mM NaCl gradient. (a-b) From left to right n = 10, 12, 14, 14, 12, 12, 14, 11 animals per condition. **(c)** Mean speed of individual worms and **(d)** the fraction of time in each behavioral state when worms are navigating a plate without NaCl. From left to right n = 7, 7, 7, 7, 7, 7, 5, 7 animals per condition. Analysis done using a 5-min time interval immediately after worms are put on the assay plate.

Statistical comparison in (a-c) by two-way ANOVA followed by Tukey's HSD post-test, and Chi-square test on binned data for (d). n.s. not significant; *$p \leq 0.05$; **$p \leq 0.01$; ***$p \leq 0.001$. Boxplots show medians, 25th and 75th percentiles as box limits. The whiskers extend to the most extreme data points not considered as outlier.

References Supplementary Information

1. Lin, C. H. A. *et al.* Insulin Signaling Plays a Dual Role in *Caenorhabditis elegans* Memory Acquisition and Memory Retrieval. *J. Neurosci.* **30**, 8001–8011 (2010).

2. Chalasani, S. H. *et al.* Neuropeptide feedback modifies odor-evoked dynamics in *Caenorhabditis elegans* olfactory neurons. *Nat. Neurosci.* **13**, 615–621 (2010).

3. Chou, J. H., Bargmann, C. I. & Sengupta, P. The *Caenorhabditis elegans odr-2* gene encodes a novel Ly-6-related protein required for olfaction. *Genetics* **157**, 211–24 (2001).

4. Altun-Gultekin, Z. *et al.* A regulatory cascade of three homeobox genes, *ceh-10*, *ttx-3* and *ceh-23*, controls cell fate specification of a defined interneuron class in *C. elegans*. *Development* **128**, 1951–69 (2001).

5. Collet, J., Spike, C. A., Lundquist, E. A., Shaw, J. E. & Herman, R. K. Analysis of *osm-6*, a gene that affects sensory cilium structure and sensory neuron function in *Caenorhabditis elegans*. *Genetics* **148**, 187–200 (1998).

6. Lai, T. & Garriga, G. The conserved kinase UNC-51 acts with VAB-8 and UNC-14 to regulate axon outgrowth in *C. elegans*. *Development* **131**, 5991–6000 (2004).

7. Tobin, D. M. *et al.* Combinatorial expression of TRPV channel proteins defines their sensory functions and subcellular localization in *C. elegans* neurons. *Neuron* **35**, 307–18 (2002).

8. Yu, S., Avery, L., Baude, E. & Garbers, D. L. Guanylyl cyclase expression in specific sensory neurons: a new family of chemosensory receptors. *Proc. Natl. Acad. Sci. U. S. A.* **94**, 3384–7 (1997).

9. Sagasti, A., Hobert, O., Troemel, E. R., Ruvkun, G. & Bargmann, C. I. Alternative olfactory neuron fates are specified by the LIM homeobox gene *lim-4*. *Genes Dev.* **13**, 1794–806 (1999).

10. Jang, H. *et al.* Dissection of neuronal gap junction circuits that regulate social behavior in *Caenorhabditis elegans*. *Proc. Natl. Acad. Sci.* **114**, E1263–E1272 (2017).

11. Glauser, D. A. *et al.* Heat avoidance is regulated by transient receptor potential (TRP) channels and a neuropeptide signaling pathway in *Caenorhabditis elegans*. *Genetics* **188**, 91–103 (2011).

12. Vidal-Gadea, A. *et al.* Magnetosensitive neurons mediate geomagnetic orientation in *Caenorhabditis elegans*. *Elife* **4**, (2015).

13. Juozaityte, V. *et al.* The ETS-5 transcription factor regulates activity states in *Caenorhabditis elegans* by controlling satiety. *Proc. Natl. Acad. Sci. U. S. A.* **114**, E1651–E1658 (2017).

14. Sarafi-Reinach, T. R. & Sengupta, P. The forkhead domain gene *unc-130* generates chemosensory neuron diversity in *C. elegans*. *Genes Dev.* **14**, 2472–2485 (2000).
