# Supplementary material for: Neuromedin U signaling regulates retrieval of learned salt avoidance in a C. elegans gustatory circuit
Source: Nat Commun. 2020 Apr 29;11:2076. doi: 10.1038/s41467-020-15964-9 (PMC7190830; doi:10.1038/s41467-020-15964-9)
Supplement: Supplementary file 3 — Description of Additional Supplementary Files [file 41467_2020_15964_MOESM3_ESM.docx]

Description of Additional Supplementary Files

**File Name:** Supplementary Data 1

**Description:** Strains, reagents and software used in this study.

**File Name:** Supplementary Data 2

**Description:** Source data for Fig. 1-7 and Supplementary Fig. 1-10
