# Supplementary material for: Neuromedin U signaling regulates retrieval of learned salt avoidance in a C. elegans gustatory circuit
Source: Nat Commun. 2020 Apr 29;11:2076. doi: 10.1038/s41467-020-15964-9 (PMC7190830; doi:10.1038/s41467-020-15964-9)
Supplement: Supplementary file 6 — Reporting Summary [file 41467_2020_15964_MOESM6_ESM.pdf]

nature research

Corresponding author(s): Dr. Isabel Beets

Last updated by author(s): Mar 30, 2020

Reporting Summary

Nature Research wishes to improve the reproducibility of the work that we publish. This form provides structure for consistency and transparency in reporting. For further information on Nature Research policies, see Authors & Referees and the Editorial Policy Checklist .

Statistics

For all statistical analyses, confirm that the following items are present in the figure legend, table legend, main text, or Methods section.

n/a	Confirmed	
☐	☒	The exact sample size (*n*) for each experimental group/condition, given as a discrete number and unit of measurement
☐	☒	A statement on whether measurements were taken from distinct samples or whether the same sample was measured repeatedly
☐	☒	The statistical test(s) used AND whether they are one- or two-sided<br>*Only common tests should be described solely by name; describe more complex techniques in the Methods section.*
☒	☐	A description of all covariates tested
☐	☒	A description of any assumptions or corrections, such as tests of normality and adjustment for multiple comparisons
☐	☒	A full description of the statistical parameters including central tendency (e.g. means) or other basic estimates (e.g. regression coefficient) AND variation (e.g. standard deviation) or associated estimates of uncertainty (e.g. confidence intervals)
☐	☒	For null hypothesis testing, the test statistic (e.g. *F*, *t*, *r*) with confidence intervals, effect sizes, degrees of freedom and *P* value noted<br>*Give P values as exact values whenever suitable.*
☒	☐	For Bayesian analysis, information on the choice of priors and Markov chain Monte Carlo settings
☒	☐	For hierarchical and complex designs, identification of the appropriate level for tests and full reporting of outcomes
☒	☐	Estimates of effect sizes (e.g. Cohen's *d*, Pearson's *r*), indicating how they were calculated

Our web collection on statistics for biologists contains articles on many of the points above.

Software and code

Policy information about availability of computer code

Data collection	An overview of all computer software and code used in this study can be found in Supplementary Table 1 of the submitted manuscript.<br>- Fluorescent microscopy and calcium imaging: Metamorph Microscopy Automation and Image Analysis Software (Molecular Devices) RRID:SCR_002368<br>- Worm population tracking: StreamPix 6 Multicamera acquisition software (NorPix, Inc.) RRID:SCR_015773
Data analysis	An overview of all computer software and code used in this study is listed in Supplementary Table 1 of this study.<br>- Offline behavioral tracking and analysis was performed using customized MATLAB (R2017b, The Mathwork, Inc., RRID:SCR_001622) scripts that are based on the Parallel Worm Tracker (Ramot et al., 2008).<br>- Fluorescence intensities were extracted using custom Wolfram Mathematica 10 scripts (RRID:SCR_014448).<br>- Statistical tests were performed using common functions in MATLAB R2017b, the R Project for Statistical Computing (RRID:SCR_001905) and GraphPad Prism 5 (RRID:SCR_002798).<br>- Plotting: MATLAB-generated and fluorescent TIFF images were arranged into figures using Inkscape (RRID:SCR_014479)<br>All custom code is available upon reasonable request.

For manuscripts utilizing custom algorithms or software that are central to the research but not yet described in published literature, software must be made available to editors/reviewers. We strongly encourage code deposition in a community repository (e.g. GitHub). See the Nature Research guidelines for submitting code & software for further information.

Data

Policy information about availability of data

All manuscripts must include a data availability statement. This statement should provide the following information, where applicable:

- Accession codes, unique identifiers, or web links for publicly available datasets
- A list of figures that have associated raw data
- A description of any restrictions on data availability

The source data underlying Figs. 1-7 and Supplementary Figs. 1-10 are provided as Supplementary Data 2. Reasonable requests for data can be directed to and will be fulfilled by Liliane Schoofs (liliane.schoofs@kuleuven.be) and Isabel Beets (isabel.beets@kuleuven.be).

Field-specific reporting

Please select the one below that is the best fit for your research. If you are not sure, read the appropriate sections before making your selection.

☒ Life sciences ☐ Behavioural & social sciences ☐ Ecological, evolutionary & environmental sciences

For a reference copy of the document with all sections, see nature.com/documents/nr-reporting-summary-flat.pdf

Life sciences study design

All studies must disclose on these points even when the disclosure is negative.

Sample size	No sample-size calculations were performed before behavioral assays. For all behavioral assays, data was collected on at least two independent days, resulting in sample sizes that are consistent with those in previous publications.
Data exclusions	As behavioral assays are subject to variation from environmental factors, great care was taken to standardize experimental conditions. Although unusual, all data from one experimental day was discarded when either wild-type or control conditions from on that day failed to conform to previous observations, which commonly could be traced back to abnomalities in ambient conditions. Within succesful experiments, some data was excluded by pre-determined thresholds in the analysis workflow. In particular, short animal trajectories that fail to reach a certain pre-defined length were discarded as these emanate from tracking issues such as collisions with other animals or the edge of the behavioral arena.
Replication	Assays were repeated at least two independent times with appropriate controls, ensuring reproducibility and consistency. Independent assays were compared to each other (with no significant changes unless noted), and the data pooled for analysis as a single group.
Randomization	Animals for behavior analysis were randomly selected. For all behavioral assays, wild-type, control and experimental conditions were either run in parallel (f.i. NaCl chemotaxis and locomotion using a multi-camera setup acquiring footage simultaneously) or randomly alternated in subsequent experimental trials (f.i. Calcium-imaging).
Blinding	As behavior was quantified using custom software scripts, excluding any bias from manual scoring, the experimenters were generally not blinded to the experimental conditions.

Reporting for specific materials, systems and methods

We require information from authors about some types of materials, experimental systems and methods used in many studies. Here, indicate whether each material, system or method listed is relevant to your study. If you are not sure if a list item applies to your research, read the appropriate section before selecting a response.

Materials & experimental systems

n/a	Involved in the study	
☒	☐	Antibodies
☐	☒	Eukaryotic cell lines
☒	☐	Palaeontology
☐	☒	Animals and other organisms
☒	☐	Human research participants
☒	☐	Clinical data

Methods

n/a	Involved in the study	
☒	☐	ChIP-seq
☒	☐	Flow cytometry
☒	☐	MRI-based neuroimaging

Eukaryotic cell lines

Policy information about cell lines

Cell line source(s)	CHO-K1 + Gα16 Parental Aequorin Cell Line (Purchased from PerkinElmer, Inc. Product number ES-000-A24. RRID:CVCL_5I89)
Authentication	Quality control and authentication of the commercial CHO cells was performed by determining the EC50 for reference agonists (e.g. ATP) in an AequoScreen calcium mobilization assay
Mycoplasma contamination	CHO cells tested negative for mycoplasma contamination, which is regularly checked using the MycoAlertTM detection kit (Lonza).
Commonly misidentified lines (See ICLAC register)	This study did not use commonly misidentified cell lines.

Animals and other organisms

Policy information about studies involving animals; ARRIVE guidelines recommended for reporting animal research

Laboratory animals	Young adult hermaphrodite C. elegans worms were used for all experiments. An overview of all used C. elegans strains can be found in Supplementary Table 1.
Wild animals	This study did not use animals captured in the wild, but common C. elegans strains bred under laboratory conditions.
Field-collected samples	No C. elegans worms were collected in the field.
Ethics oversight	No ethical approval was needed for this work as the invertebrate nematode C. elegans is not a sentient animal model.

Note that full information on the approval of the study protocol must also be provided in the manuscript.
